# Chronological and biological age: Why relevant for psychiatrists?

**DOI:** 10.1192/j.eurpsy.2021.194

**Published:** 2021-08-13

**Authors:** B. Penninx

**Affiliations:** Psychiatry, Amsterdam UMC, Vrije Universiteit, Amsterdam, Netherlands

**Keywords:** aging, biological aging, depression

## Abstract

**Introduction:**

Depression is the mental disorder with the largest disease burden impact. That is due to its high prevalence, chronicty, early onset but also due to its impact on various aging-related somatic morbidities and mortality. This talk will describes to what extent depression characteristics are related to chronologial and biological aging patterns.

**Methods:**

Data will be shown from the Netherlands Study of Depression and Anxiety (NESDA, www.nesda.nl). In this study, a large cohort of over 3000 individuals (18-65 years), among which over 1200 with a DSM-based major depressive disorder (MDD), are now followed for 9 years. The association between depression characteristics and chronological and biological age will be described. Biological age was determined at various biological system-levels, including telomere length, epigenetics, transcriptomics, metabolomics and proteomics.

**Results:**

Older persons with a current MDD do not differ in overall disease severity as compared to younger persons with a current MDD. However, older depressed persons do differ in the types of symptoms they experience (more neurovegetative, somatic symptoms and less mood symptoms) and in their chronic course (with twice more chronicity in the oldest depressed persons compare to the younges depressed persons). At all biological system-levels, there was evidence for more advaned biological aging among persons with depression. This was not differential across chronological age groups. Discussion: Findings suggest that depression characteristics are linked to both chronological and biological age. It will be discussed what this could mean for clinical practice and intervention.

**Disclosure:**

No significant relationships.

